# Alcohol’s Burden on Immunity Following Burn, Hemorrhagic Shock, or Traumatic Brain Injury

**DOI:** 10.35946/arcr.v37.2.10

**Published:** 2015

**Authors:** Patricia E. Molina, Paige S. Katz, Flavia Souza-Smith, Stephen M. Ford, Sophie X. Teng, Tracy Y. Dodd, John K. Maxi, Jacques P. Mayeux

**Affiliations:** Patricia E. Molina, M.D., Ph.D., is a professor and the department head; Paige S. Katz, Ph.D., is a postdoctoral fellow; Flavia Souza-Smith, Ph.D., is an instructor; Stephen M. Ford is a graduate student; Sophie X. Teng, Ph.D., is a medical student; Tracy Y. Dodd, Ph.D., is a postdoctoral fellow; John K. Maxi is a graduate student; Jacques P. Mayeux, M.S., is a graduate student; all in the Department of Physiology, Comprehensive Alcohol Research Center and Alcohol and Drug Abuse Center of Excellence, Louisiana State University Health Sciences Center, New Orleans, Louisiana.

**Keywords:** Alcohol consumption, alcohol use, abuse, and dependence, chronic alcohol use, acute alcohol use, injury, traumatic injury, morbidity, mortality, immune response, impaired immune response, bacterial pathogens, viral pathogens, tissue, organs, disease

## Abstract

Alcohol consumption contributes to increased incidence and severity of traumatic injury. Compared with patients who do not consume alcohol, alcohol-consuming patients have higher rates of long-term morbidity and mortality during recovery from injury. This can be attributed in part to an impaired immune response in individuals who consume alcohol. Acute and chronic alcohol use can affect both the innate and adaptive immune defense responses within multiple organ systems; the combination of alcohol use and injury results in increased susceptibility to bacterial and viral pathogens. This review examines the major deleterious effects of alcohol on immunity following tissue damage or traumatic injury, with a focus on alcohol’s influence on the ability of the immune and major organ systems to fight disease and to repair damaged tissues following injury.

The incidence of traumatic injury in alcohol-intoxicated individuals continues to escalate. According to the Centers for Disease Control and Prevention (2012*a*), more than 38 million American alcohol users consume 5 or more drinks on the same occasion (i.e., binge drink) and do so about 4 times per month. This behavior is highly conducive to unintentional or accidental traumatic injury, which according to the National Vital Statistics Reports is the leading cause of years of potential life lost (YPLL) before age 45. Unintentional injury causes more YPLL than that attributed to cancer, intentional injuries, heart disease, and HIV individually (Centers for Disease Control and Prevention 2009). Data from the National Center for Injury Prevention and Control, as well as data derived from prospective and retrospective studies, show that up to 40 percent of victims of traumatic injury have positive blood alcohol concentrations (BAC), with 35 percent presenting with blood alcohol levels above the legal limit of intoxication (Beech and Mercadel 1998).

The severity of trauma, reduced blood flow and oxygen delivery (i.e., hemorrhagic shock, referred to as shock in this article), and tissue injury is greater in intoxicated victims than in sober victims, resulting in higher mortality rates in the alcohol-consuming patient population (Pories et al. 1992). Although immediate mortality from traumatic injury has improved significantly as a result of aggressive resuscitation, long-term morbidity and mortality continue to be unacceptably high during the recovery period. The prevalence of morbidity and mortality is particularly attributable to the altered immune response among impaired patients to subsequent challenges, such as surgery or infection, leading to multiple organ failure (Roumen et al. 1993; Sauaia et al. 1994). Acute alcohol intoxication complicates the initial management of trauma victims and is associated with greater incidences of pneumonia and respiratory distress, requiring ventilator assistance during hospitalization (Gurney et al. 1992; Jurkovich et al. 1992). In addition, major complications including tracheobronchitis, pneumonia, pancreatitis, and sepsis are significantly increased in patients with high levels of carbohydrate-deficient transferrin (CDT), a marker for alcoholism (Spies et al. 1998). European studies show that, compared with nonalcoholics, alcoholics more frequently develop major complications and require a significantly prolonged stay in the intensive care unit (ICU) following trauma (Spies et al. 1996*a*).

Excessive acute and chronic alcohol consumption has significant effects at multiple cellular levels, affecting both innate and adaptive immune mechanisms (Molina et al. 2010). Both chronic and acute patterns of alcohol abuse lead to impaired immune responses, resulting in increased susceptibility to infectious diseases caused by bacterial and viral pathogens (Brown et al. 2006). Clinical and preclinical studies show that the combined effects of alcohol and injury result in greater immune disruption than either insult alone (Messingham et al. 2002). This article reviews the current understanding of the burden of alcohol on the immune response to three specific traumatic events: burn, shock, and traumatic brain injury (TBI). The major pathophysiological consequences of these injuries on other major organ systems— including the cardiovascular system, pulmonary system, and gastrointestinal tract—are highlighted with emphasis on the contribution of alcohol-induced immunomodulation to postinjury morbidity.

Reestablishment of homeostasis after a traumatic insult involves activation of host defense mechanisms for self-protection against toxic inflammatory processes and tissue repair. Trauma victims frequently are subjected to necessary invasive procedures, such as surgery and anesthesia. In addition, trauma victims frequently are exposed to subsequent challenges, particularly infection. These additional stresses to an already compromised inflammatory and neuroendocrine milieu further contribute to morbidity and mortality in this patient population. Traumatic injury and hemorrhagic shock produce a temporal pattern with early upregulation of pro-inflammatory cytokine1 gene product expression and with later suppression of stimulated pro-inflammatory cytokine release (Hierholzer et al. 1998; Molina et al. 2001). Together, these alterations lead to generalized immunosuppression, ultimately resulting in an increased susceptibility to infection (Abraham 1993; Ertel et al. 1993).

Alcohol has been shown to affect multiple aspects of the host immune response, contributing to pathological processes (Szabo 1998). For example, alcohol alters the expression and processing of cytokines and a type of cytokine known as chemokines (D’Souza et al. 1989; Standiford and Danforth 1997), the expression of adhesion molecules (Zhang et al. 1999), inflammatory cell recruitment (Patel et al. 1996; Shellito and Olariu 1998) and accumulation, and oxidative capacity of macrophages (Nilsson and Palmblad 1988). The monocyte/macrophage production of cytokines and chemokines, in particular interleukin (IL)-8 and tumor necrosis factor-α (TNF-α), is critical in the regulation of the acute inflammatory host response to infectious challenge. The combined inhibition of pro-inflammatory cytokine production and neutrophil activation and migration to a site of infection has been suggested to contribute to the enhanced susceptibility to infection in alcoholic individuals (Nelson et al. 1991) and to the increased risk of trauma- and burn-related infections associated with alcohol intoxication (Arbabi et al. 1999). Several lines of evidence show that these alcohol-mediated alterations in host defense following injury lead to increased morbidity and mortality from infections during the recovery period (Faunce et al. 2003; Messingham et al. 2002; Zambell et al. 2004). In addition, considerable evidence suggests that the severity of disease processes is greater in intoxicated trauma victims than in nonintoxicated counterparts (Spies et al. 1996*a*,*b*, 1998). In particular, immunoparalysis characterized by inhibition of stimulated pro-inflammatory cytokine release (Angele et al. 1999) and alterations of both cellular and humoral immunity (Napolitano et al. 1995; Wichmann et al. 1998) have been identified as risk factors for infection and progression to organ injury during the posttraumatic injury period (Abraham 1993; Ertel et al. 1993).

The systemic response to injury is associated with marked activation of neuroendocrine pathways that contribute to cardiovascular adaptation to blood loss, injury, and pain but also exert immunomodulatory effects (Molina 2005). Catecholamines (e.g., dopamine, norepinephrine, and epinephrine), and drugs that mimic their effects (i.e., adrenergic agonists), are especially known to exert important regulatory functions on macrophages as well as on B- and T-lymphocyte cytokine production, proliferation, and antibody secretion; dendritic cell function; cytokine and chemokine release; and nitric oxide (NO) production (Madden et al. 1995). The relevance of these control mechanisms and the implications of their dysregulation have been demonstrated by the high incidence of infection in patients who experience elevated temperature, increased heart rate, and perspiration (i.e., “sympathetic storm”) following acute brain trauma and myocardial infarction (Woiciechowsky et al. 1998). Alcohol intoxication produces marked disruption of several neuroendocrine pathways. Disruption of the homeostatic neuroendocrine counterregulatory response to shock impairs hemodynamic stability and recovery, contributing to compromised blood flow and increased end-organ injury (Molina et al. 2013). Specifically, binge alcohol use blunts central neuroendocrine and autonomic activation, and this seems to result from alcohol-accentuated NO production in the periventricular nucleus (PVN) of the hypothalamus (Whitaker et al. 2010). Alcohol-mediated impairment of neuroendocrine counterregulatory responses to traumatic injury not only exacerbates low blood pressure (i.e., hypotension) during hemorrhage but also attenuates blood pressure recovery during fluid resuscitation, leading to significant alterations in blood flow redistribution and notably affecting circulation in the gastrointestinal tract (Wang et al. 1993). Studies have shown that alcohol-intoxicated animals have greater reduction of blood flow to the liver, kidney, and small and large intestines than nonintoxicated animals, following shock and fluid resuscitation (Sulzer et al. 2013). These macro- and microcirculatory changes during trauma and hemorrhage have been implicated in the subsequent development of sepsis and multiple organ failure (Peitzman et al. 1995) and contribute to an increased host susceptibility to infection and tissue injury during recovery (Mathis et al. 2006; Xu et al. 2002). People who abuse alcohol, including both binge and chronic drinkers, have a higher incidence of traumatic injury such as burn, shock, and TBI. The host response to these diverse insults is markedly affected by both patterns of alcohol abuse and some systems—including gastrointestinal, cardiovascular, and pulmonary— are more affected than others according to the specific injury.

## Alcohol and Burn Injury

**Figure 1:**
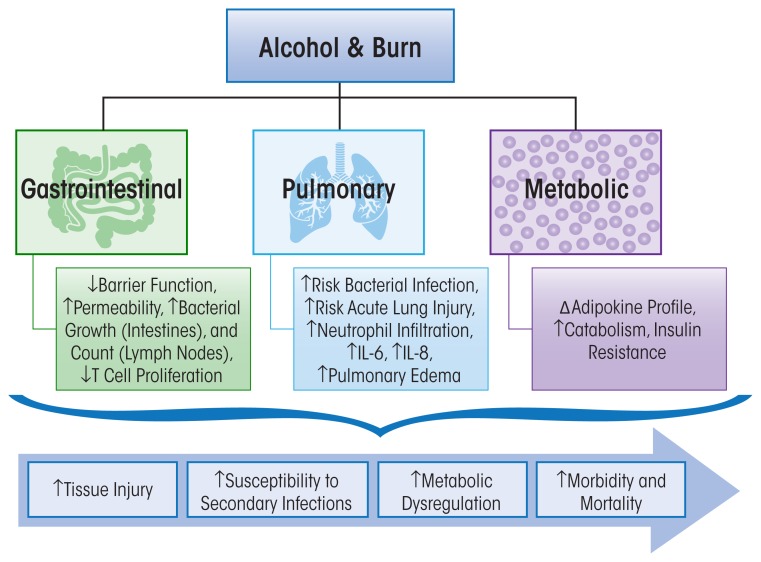
Salient gastrointestinal, pulmonary, and metabolic pathophysiological consequences of alcohol abuse prior to, or at the time of, burn injury. The decrease in gut barrier function leads to increased permeability and bacterial translocation that enhances the risk for bacterial infections and lung injury. Marked alterations in metabolic responses, characterized by altered adipokine profile consistent with increased insulin resistance, collectively contribute to greater morbidity and mortality post–burn injury.

Burn injury is a common type of traumatic injury that affects thousands of people in the United States every year (Bessey et al. 2014). Approximately 50 percent of burn-injured patients have detectable blood alcohol levels at the time of hospital admission (Haum et al. 1995; McGwin et al. 2000), and these patients have more complications, require longer hospital stays, and have greater mortality rates than those with a similar degree of injury who are not intoxicated at the time of injury (McGill et al. 1995). Most morbidity and mortality among patients who survive initial injury is attributed to complications stemming from infection (Baker et al. 1980). Therefore, the pre-burn immunological condition of injured patients affects susceptibility to infection and survival. Several mechanisms contribute to infection in burn patients, including loss of barrier function, changes in normal flora, wound ischemia, and cellular immunosuppression resulting from pro-inflammatory processes. Neutrophil, helper T-cell, and macrophage dysfunction; increased pro-inflammatory cytokine production; and enhanced production of immunosuppressive factors have all been shown to contribute to the pathophysiological response to burn injury (Faunce et al. 1998; Messingham et al. 2000). The mechanisms that contribute to infection in burn patients are influenced by acute and chronic alcohol intoxication and will be discussed below (see figure 1).

Research by Kovacs and colleagues (2008) has offered insight into the combined effects of burn injury and alcohol intoxication on immunity (Bird and Kovacs 2008). Chronic alcohol abuse alone increases the risk for lung infection (Baker and Jerrells 1993), impairs the phagocytic activity of alveolar macrophages and clearance of infectious particles from the airways, and impairs oxidant radicals, chemokine, and cytokine release that are required for microbial killing (Brown et al. 2007; Mehta and Guidot 2012; Molina et al. 2010). Acute alcohol intoxication prior to burn injury significantly suppresses the immune response relative to the insult alone (Faunce et al. 1997) and causes greater suppression of T-cell proliferation and response, reduced IL-2 production, and increased IL-6 production and circulating levels (Choudhry et al. 2000; Faunce et al. 1998). The T-cell and cytokine impairment caused by the combined effect of alcohol and burn injury may further suppress cell-mediated immunity, resulting in even greater susceptibility to infection than burn alone. Alcohol-mediated immunomodulation contributes to tissue injury in target organs as described below.

### Gastrointestinal Tract

A multitude of studies have demonstrated that the gut is a reservoir for pathogenic bacteria, which may contribute to increased susceptibility to infections following traumatic injury (Deitch 1990). The intestinal mucosal barrier serves a major role in the local defense against bacterial entry and the translocation of endotoxin to the systemic circulation (Xu et al. 1997). Increased permeability and immune dysfunction indicate the compromised state of the intestinal mucosal barrier to bacterial translocation following trauma (Deitch et al. 1990; Willoughby et al. 1996). Increased intestinal permeability enhances bacterial and endotoxin translocation from the intestinal tract to the systemic circulation, triggering a systemic inflammatory response (Xu et al. 1997). Activated macrophages and lymphocytes release pro-inflammatory cytokines including TNF-α, IL-1β, and IL-6, thereby contributing to tissue injury (Fink 1991). Studies have determined that chronic alcohol consumption disrupts intestinal barrier function and induces gut leak (Li et al. 2008; Tang et al. 2009). In addition, reports have shown a loss of intestinal barrier function followed by an increase in endotoxin and bacterial translocation to the systemic circulation following burn injury alone (Carter et al. 1990; Deitch and Berg 1987; Horton 1994), alcohol intoxication alone (Keshavarzian et al. 1994; Tabata et al. 2002), and burn injury with alcohol intoxication (Choudhry et al. 2002; Kavanaugh et al. 2005; Napolitano et al. 1995). Acute alcohol intoxication at the time of burn injury enhances bacterial growth in the intestine and is reflected in a proportional increase in mesenteric lymph node bacterial count (Kavanaugh et al. 2005). Acute alcohol intoxication also modulates intestinal immune defense by suppressing T-cell proliferation and increasing bacterial accumulation in mesenteric lymph nodes, spleen, and blood, which suggests that T-cell suppression may play a role in bacterial translocation from the lumen of the gut (Choudhry et al. 2002). Moreover, studies have shown that following shock, trauma, or burn injury, the gut leaks bacteria and pro-inflammatory factors that are carried by the mesenteric lymphatic system, which contributes to acute lung injury (ALI) (Magnotti et al. 1999). The possibility that alcohol exacerbates toxin delivery to the systemic circulation through the lymphatics is supported by studies demonstrating that alcohol regulates the contractile cycle of mesenteric lymphatic vessels modulating the driving force of lymph flow (Keshavarzian et al. 1994; Souza-Smith et al. 2010). Thus, the contribution of gut–lymph to end-organ damage following burn injury and alcohol intoxication may be significant.

Collectively, studies indicate that alcohol consumption preceding burn injury (1) increases gut permeability; (2) enhances intestinal bacterial growth, translocation, and systemic accumulation; and (3) suppresses T-cell proliferation. Further, research supports the concept that the intestine is not only a source of infection but also the site of the initial immune perturbation leading to the development of multiple organ dysfunction or organ failure.

### Cardiovascular System

Immediately following a burn injury, the cardiovascular system responds with a decrease in cardiac output (Cuthbertson et al. 2001) as a result of low blood volume and reduced venous return (Kramer et al. 2007). This phase is associated with decreased cardiac contractility, mediated by the release of vasoactive and pro-inflammatory mediators (Williams et al. 2011). Subsequently, there is a surge in counterregulatory neuroendocrine mediators (catecholamines, glucagon, and cortisol) that contribute to the development of a hyperdynamic cardiovascular state—characterized by increased heart rate and cardiac output—and is associated with increased myocardial oxygen consumption and myocardial hypoxia (Williams et al. 2011). These pathophysiological processes enhance oxidative metabolism and increase the risk for free-radical generation, further exacerbating the pro-oxidative environment that has been proposed to contribute to impaired wound healing in burn patients (Herndon and Tompkins 2004). Chronic binge alcohol consumption also has been shown to promote a pro-oxidative and pro-inflammatory milieu (Rashbastep et al. 1993), and these factors may further impede wound healing in patients consuming alcohol prior to experiencing burn injury. Additional research is needed to better understand immunomodulation effects following the combined insults of alcohol and burn injury and the mechanisms underlying the more severe outcome of burn injury with alcohol abuse.

### Pulmonary System

Adult respiratory distress syndrome (ARDS) is a frequent cause of death in burn patients. The lungs are one of the first organs to fail following traumatic injury (Turnage et al. 2002). Chronic and acute alcohol abuse impair pulmonary host defense to infection, thus increasing the risk of bacterial infection and acute lung injury (Boe et al. 2009; Happel and Nelson 2005). Lung injury as a result of the combination of alcohol intoxication and burn injury may be attributed to the delicate architecture of the lungs combined with other alcohol-related factors, such as bacterial and endotoxin leakage from the gut and a higher risk of contact with pathogens from the circulation and airways (Bird and Kovacs 2008; Li et al. 2007). Previous studies show that the combined insult of acute alcohol consumption and burn injury in mice leads to increased infiltration of the lungs by white blood cells, called neutrophils, and pro-inflammatory cytokine expression of IL-6 (Chen et al. 2013). Systemic and pulmonary IL-6 reflect the inflammatory state of the host and have been shown to be decreased in the absence of Toll-like receptor-4 (TLR-4) and intercellular adhesion molecule-1 (ICAM-1) (Bird et al. 2010). The role of IL-6 in lung injury has been demonstrated in studies in IL-6 knockout mice or following neutralization of IL-6, both of which result in significantly reduced lung inflammation (Chen et al. 2013). Studies also have shown that acute alcohol intoxication at the time of burn injury induces an upregulation of IL-18 production and neutrophil infiltration within the lung compartment, all leading to pulmonary edema (Li et al. 2007).

### Metabolism

The post-burn period is characterized by a hypermetabolic state (Pereira and Herndon 2005) consisting of increased oxygen consumption; increased breakdown of glycogen, fats, and proteins; elevated resting energy expenditure and glucose synthesis; and reduced insulin-stimulated glucose uptake into skeletal muscle and adipose tissue (Gauglitz et al. 2009). Previous studies suggest that development of this hypermetabolic state during the post-burn period occurs as a consequence of (1) increased plasma catecholamine and corticosteroid concentrations (Jeschke et al. 2008; Williams et al. 2009; Wilmore and Aulick 1978), (2) increased systemic pro-inflammatory mediator expression, favoring processes that release energy (i.e., catabolic) over those that store energy (i.e., anabolic) (Jeschke et al. 2004), and (3) increased adipose tissue mRNA (Zhang et al. 2008) and protein (Yo et al. 2013) expression of uncoupling protein-1 (UCP-1), enhancing heat production and metabolism. Further, circulating levels of TNF-α, a known anti-insulin cytokine, are increased (Keogh et al. 1990), and the post-burn period can be described as a state of marked insulin resistance (IR) (Gauglitz et al. 2009). Insulin sensitivity has been reported to be decreased by more than 50 percent at 1-week post–burn injury in pediatric patients (Cree et al. 2007) as well as in rodent models of burn injury (Carter et al. 2004). The relevance of insulin levels to overall outcome from burn injury is supported by results from clinical studies showing that exogenous insulin therapy in pediatric burn patients decreased pro-inflammatory cytokines, increased anti-inflammatory cytokines, and increased serum concentrations of insulin-like growth factor-1 (IGF-1) and insulin-like growth factor binding protein-3 (IGFBP-3). Together, these changes could help to preserve organ function and better promote anabolic processes during the post-burn hypermetabolic state (Jeschke et al. 2004). Chronic alcohol consumption decreases insulin responsiveness and can alter insulin signaling through various mechanisms, including increased hepatic protein expression of the gene phosphatase and tensin homologue (PTEN), which directly inhibits insulin signaling through the phosphatidylinositol-5,5-bisphosphate 3-kinase (PI3K)/protein kinase B (Akt) pathway (de la Monte et al. 2012). In addition to the negative regulation of the pathway by PTEN proteins, the enzyme protein tyrosine phosphatase dephosphorylates and decreases activity of important molecules involved in the insulin signaling cascade, potentially contributing to impaired insulin action (Gao et al. 2010; Koren and Fantus 2007). In addition, Lang and colleagues (2014) demonstrated that chronic alcohol consumption reduces Akt and AS160 phosphorylation, reduces membrane localization of glucose transporter type 4 (GLUT-4) protein, and increases serine phosphorylation at serine-307 of insulin receptor substrate-1 (IRS-1), all of which will attenuate insulin-stimulated skeletal muscle glucose uptake and other insulin-mediated anabolic effects (Lang et al. 2014). These negative effects on insulin signaling occurred in conjunction with sustained increases in pro-inflammatory cytokines TNF-α and IL-6 following chronic alcohol exposure (Lang et al. 2014). Thus, both burn injury and chronic alcohol exposure alter metabolic pathways—favoring catabolic and opposing anabolic pathways—possibly resulting in long-lasting alterations in metabolic processes. The metabolic dysregulation following burn injury is likely to produce more severe consequences in chronic alcohol burn victims. Previous studies assessing nutritional status of alcoholic patients have been discordant, with some studies suggesting that increased alcohol consumption increases the prevalence of malnutrition in alcoholic patients (Hillers and Massey 1985), whereas other studies do not show a role for excessive, or chronic, alcohol consumption in malnutrition (Nicolas et al. 1993; Urbano-Marquez et al. 1989). A study assessing the influences of aging and chronic alcohol feeding in mice on protein synthesis demonstrated that chronic alcohol feeding decreases gastrocnemius muscle protein synthesis, which provides a mechanism for loss of lean body mass (Korzick et al. 2013; Lang et al. 2014). Decreased anabolism during the post-burn period, which itself is a state of heightened catabolic processes, could significantly impair recovery for these alcoholic patients experiencing burn injury. Further, the hypermetabolic state of the post-burn period is thought to contribute to delayed or impaired wound healing, increased susceptibility to infections, and erosion of lean body mass (Pereira and Herndon 2005). Moreover, both binge alcohol consumption (Pravdova and Fickova 2006; You and Rogers 2009) and burn injury (Venkatesh et al. 2009; Wade et al. 2013) can contribute to dysregulation of cytokines secreted by adipose tissue (i.e., adipokines). Recent studies show that mice exposed to a single alcohol binge prior to burn injury have a dramatic increase in pro-inflammatory response and a decrease in anti-inflammatory response in adipose tissue (Qin et al. 2014). The heightened pro-inflammatory response during the post-burn period would be predicted to modulate leptin levels. Thus, recovery from burn injury is likely to be severely impaired in alcoholic individuals as a result of a greater disruption in metabolic processes as well as impairment of host defense mechanisms, leading to greater morbidity and health care costs associated with the management of these patients. Therefore, further investigation is warranted to understand the modulation of the immune system by the combined effect of alcohol and burn that might result in dysregulation of adipose tissue and altered metabolism.

## Alcohol and Hemorrhagic Shock

Studies from several investigators have provided evidence that traumatic injury and hemorrhagic shock produce an immediate upregulation of pro-inflammatory cytokine gene product expression (Ayala et al. 1991; Hierholzer et al. 1998). The early pro-inflammatory response is later followed by suppression of stimulated pro-inflammatory cytokine release (Angele et al. 1999; Xu et al. 1998) and alterations of both cellular and humoral immunity (Napolitano et al. 1995; Wichmann et al. 1998), leading to generalized immunosuppression, which ultimately results in an increased susceptibility to infection (Abraham 1993; Ertel et al. 1993). Along with marked alterations in hemodynamic homeostasis and neuroendocrine regulation, immunological derangements and subsequent infections are also a major cause of increased morbidity and mortality following hemorrhagic shock (Livingston and Malangoni 1988; Phelan et al. 2002).

**Figure 2:**
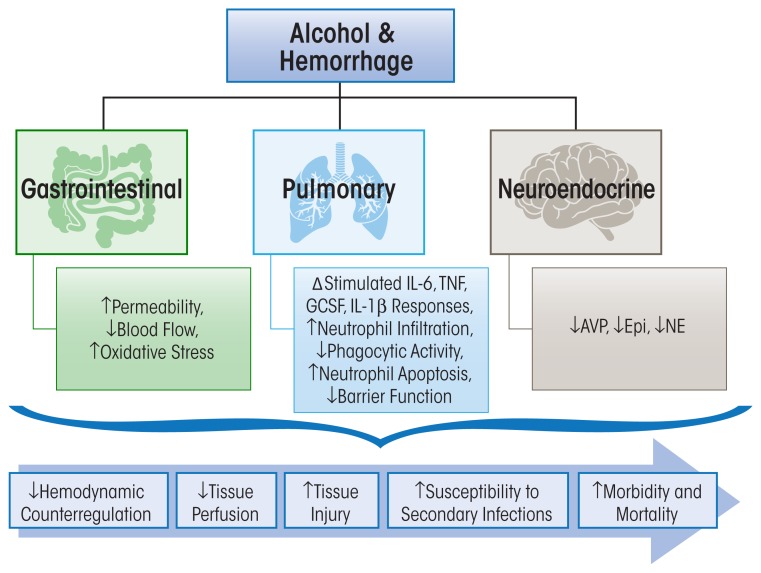
Salient gastrointestinal, pulmonary, and neuroendocrine pathophysiological consequences of alcohol abuse prior to, or at the time of, hemorrhagic shock. The decreased hemodynamic counterregulatory response leads to decreased tissue perfusion, accentuated oxidative stress, and enhanced tissue injury. In addition, the alcohol/hemorrhaged host shows greater susceptibility to secondary infections leading to increased morbidity and mortality during the post-injury period.

Studies focused on the immune modulatory effects of alcohol exposure following hemorrhagic shock have demonstrated that even 24 hours after the post-hemorrhagic shock, alcohol-intoxicated animals had a marked suppression in cytokine release to an inflammatory challenge (Greiffenstein et al. 2007), affecting the ability to fight secondary infectious challenges. Conversely, findings observed at the tissue level determined that alcohol intoxication enhanced the pro-inflammatory milieu following hemorrhagic shock, priming tissues for injury. The burden of alcohol and hemorrhagic shock on specific target organ systems is discussed below and summarized in figure 2.

### Gastrointestinal Tract

Hemorrhagic shock produces similar alterations in gut barrier function to those resulting from burn injury. Alcohol intoxication at the time of hemorrhagic shock further exacerbates hemorrhagic injury-induced gut permeability and leakage (Sulzer et al. 2013). Chronic alcohol consumption has been shown to disrupt intestinal barrier function and induce gut leak (Li et al. 2008; Tang et al. 2009). The combination of greater hypotension and inadequate tissue blood flow (i.e., hypoperfusion) observed in alcohol-intoxicated animals and the increased gut leak observed in alcohol-intoxicated hemorrhaged animals are speculated to contribute to increased host susceptibility to infection and tissue injury during recovery (Molina et al. 2013). Alcohol-intoxicated, hemorrhaged animals have been shown to have greater reduction in hepatic, renal, and intestinal blood flow than that observed in nonintoxicated animals (Sulzer et al. 2013). This reduction in critical organ blood flow was associated with enhanced tissue damage. An additional mechanism that could contribute to tissue injury in the alcohol-intoxicated, hemorrhaged host is the disruption of gut-associated lymphoid tissue function, which has been shown to play a role in other disease states.

### Cardiovascular System

Studies using a rodent model of binge-like alcohol consumption prior to hemorrhagic shock have shown that acute alcohol intoxication decreases basal mean arterial blood pressure (MABP), exacerbates hypotension, and attenuates blood pressure recovery during fluid resuscitation (Mathis et al. 2006; Phelan et al. 2002). Following fixed-volume hemorrhage, alcohol-intoxicated animals were significantly more hypotensive throughout the hemorrhage and resuscitation periods (Mathis et al. 2006). In response to a fixed-pressure (40 mmHg) hemorrhage, a significantly lesser amount of blood was removed from the alcohol-intoxicated animals than controls (Phelan et al. 2002). Similarly, McDonough and colleagues, using a guinea pig model of ethanol exposure prior to hemorrhagic shock (loss of 60% blood volume) and resuscitation, demonstrated that a low dose of ethanol (1 g/kg) decreases MABP and heart rate and exacerbates the metabolic effects of hemorrhagic shock, as shown by increased glucose and lactate concentrations (McDonough et al. 2002). Despite the plethora of previous studies that have examined functional cardiovascular consequence of hemorrhagic shock and hemorrhage with alcohol intoxication, few studies have examined the combined effects of alcohol, hemorrhagic shock, and immune dysfunction on the cardiovascular system. However, exacerbation of pre-existing cardiovascular disease and prolonged recovery are anticipated outcomes of the combined effects of alcohol and hemorrhagic shock, all leading to an impaired immune response.

### Pulmonary System

As mentioned previously, alcohol intoxication produces significant dysregulation of the host defense mechanism during the post-injury period. Lung IL-6 and TNF-α are suppressed, while granulocyte-colony stimulating factor (GCSF) mRNA is increased in alcohol-intoxicated, hemorrhaged animals (Mathis et al. 2006; Ono et al. 2004). Moreover, isolated pleural cells and peripheral blood mononuclear cells (PBMCs) from alcohol-intoxicated, hemorrhaged animals display suppressed TNF-α, IL-1β, and IL-6 release following lipopolysaccharide stimulation (Greiffenstein et al. 2007), suggesting greater impairment of humoral immune response than that resulting from hemorrhagic shock alone. The importance of these alterations in host defense mechanisms was demonstrated in animals inoculated with *Klebsiella pneumonia* following hemorrhagic shock. These studies showed suppressed neutrophil response, decreased phagocytic activity, and increased neutrophil apoptosis in hemorrhaged animals that were alcohol intoxicated at the time of injury (Zambell et al. 2004). This was associated with greater lung bacterial counts and prolonged elevation in TNF-α and IL-6 levels (18 h) post-infection. Furthermore, only 30 percent of alcohol-intoxicated, hemorrhaged animals survived compared with 70 percent survival of dextrose/hemorrhage animals (Zambell et al. 2004). In addition to cytokine dysregulation, alcohol impairs innate barrier functions of the lung by increasing epithelial cell permeability and altering the function of the ciliated epithelium (Elliott et al. 2007; Molina et al. 2010).

### Neuroendocrine System

The pathophysiology of traumatic-hemorrhagic injury involves decreased blood volume (i.e., hypovolemia) and hypoperfusion, which results in signaling to central cardiovascular centers aimed at restoring hemodynamic stability through activation of descending autonomic neuroendocrine pathways (Molina 2005). Several mechanisms have been proposed to account for the increased hypotension and impaired hemodynamic stability observed with alcohol intoxication, with one proposed mechanism being blunted neuroendocrine activation. Studies demonstrated that acute alcohol intoxication at the time of injury results in significant attenuated release of counterregulatory hormones and potent vasoconstrictors such as arginine vasopressin (AVP), epinephrine, and norepinephrine in response to fixed-pressure hemorrhage (Phelan et al. 2002). A disruption in the neuroendocrine response with alcohol intoxication at the time of injury is associated with enhanced expression of lung and spleen TNF-α as well as suppression of circulating neutrophil function, which would be expected to enhance the risk for tissue injury (Whitaker et al. 2010). Conversely, Sato and colleagues (2013) demonstrated that alcohol aggravates hemorrhagic shock in a dose-dependent manner not by triggering an immune response but by suppressing hormonal and neuro-humoral responses, thereby inhibiting hemodynamic auto-regulation and shortening the survival interval. Thus, both alcohol and hemorrhagic shock have detrimental effects on neuroendocrine responses that are likely to modulate the host immune system in addition to impacting on hemodynamic stability and recovery and accentuating tissue hypoperfusion and end-organ injury.

## Alcohol and Traumatic Brain Injury

**Figure 3:**
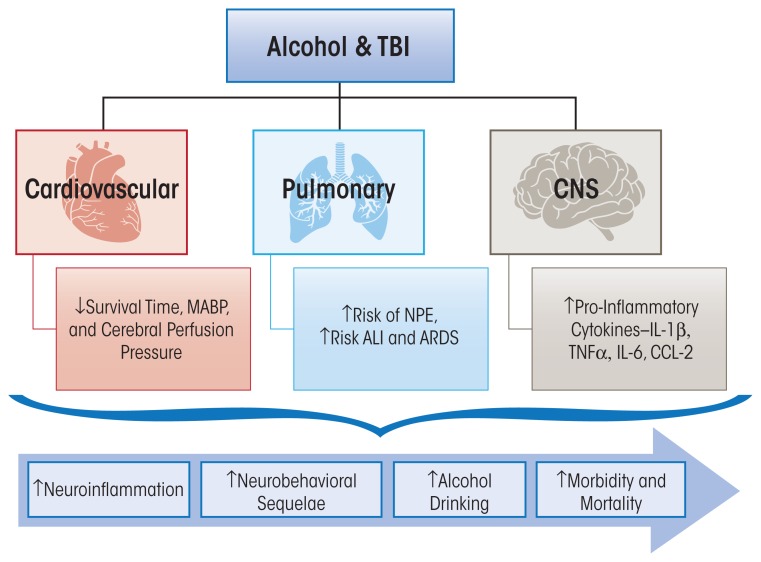
Salient cardiovascular, pulmonary, and central nervous system pathophysiological consequences of alcohol abuse prior to, or at the time of, traumatic brain injury (TBI). The disruption in hemodynamic homeostasis resulting from TBI contributes to decreased cerebral perfusion pressure. The lung is affected through neurogenic mechanisms leading to neuropulmonary edema (NPE) and associated risk for acute lung injury (ALI) and adult respiratory distress syndrome (ARDS). In the brain (CNS), alcohol accentuates neuroinflammation, which is associated with neurobehavioral dysfunction that can potentially promote alcohol drinking. Together, these pathophysiological consequences increase morbidity and mortality from TBI.

Traumatic brain injury (TBI) accounts for approximately 50 percent of all trauma-related mortality (Centers for Disease Control and Prevention 2012*b*). TBI affects multiple sectors of the population, and young males have the highest rates of hospital visits and death (Faul et al. 2010). Falls are the first leading cause of TBI, followed by motor vehicle accidents and unintentional trauma sustained during sports activities such as football or boxing. TBI can be categorized as mild, moderate, or severe, and the majority of TBIs sustained in the United States are in the mild category (Centers for Disease Control and Prevention 2012*b*). In addition to the physical dysfunction caused by injury, TBI patients frequently experience lingering psychological symptoms, such as heightened anxiety, depression, sleep disturbances, and pain hypersensitivity (Whyte et al. 1996). These symptoms have been implicated in increased alcohol intake following TBI in humans (Adams et al. 2012). Furthermore, it is well accepted that alcohol consumption increases the risks of sustaining a TBI (Corrigan 1995; Hurst et al. 1994). Nevertheless, a comprehensive understanding of the influences of alcohol on TBI-induced inflammation, recovery from injury, and long-term damage currently is limited and is summarized in the following section (see figure 3).

### Neuroinflammation

The pathophysiology of TBI involves a primary mechanical injury followed by a secondary tissue injury resulting from neuroinflammation (Werner and Engelhard 2007). A large percentage of TBI victims show signs of further deterioration following the event (Sauaia et al. 1995). This suggests the induction of a secondary brain injury and immune activation as the key cascades contributing to the pathophysiological processes of the secondary damage (Cederberg and Siesjo 2010). After TBI, a series of events occurs, including the activation of resident immune cells such as astrocytes and microglia, release of pro-inflammatory cytokines and chemokines, upregulation of endothelial adhesion molecules, and recruitment and activation of blood-derived leukocytes across the disrupted blood brain barrier (Feuerstein et al. 1998; Morganti-Kossmann et al. 2001; Ransohoff 2002). An increase in the levels of TNF-α in the serum or cerebrospinal fluid in victims of TBI also has been detected in rodents following closed head injury (Goodman et al. 1990; Ross et al. 1994; Shohami et al. 1994). IL-1β is released after TBI (Fan et al. 1995) and induces nuclear factor-kappa B (NF-κ B), a key transcription factor that regulates the expression of genes encoding cytokines, as well as inducible NO synthase (iNOS), and cyclooxygenase-2 (COX-2) (Blanco and Guerri 2007; Woodroofe et al. 1991; Ziebell and Morganti-Kossmann 2010). Following the rise of early cytokines, the release of IL-6 is associated with increased acute-phase proteins, as well as blood–brain barrier disruption (Kossmann et al. 1995; Shohami et al. 1994; Woodcock and Morganti-Kossmann 2013) and sustained elevation of chemokines such as chemokine (C-C motif) ligand-2 (CCL-2) in the cerebrospinal fluid for as long as 10 days post-injury (Semple et al. 2010). Although early cytokine release is essential in mediating the reparative processes after injury (Ziebell and Morganti-Kossmann 2010), sustained elevation of pro-inflammatory mediators has been increasingly recognized to play a role in neuropathological changes associated with long-term degenerative diseases (Fan et al. 1995; Lyman et al. 2014). Accordingly, the additional risks of alcohol as a factor contributing to the alterations of TBI-induced neuroinflammatory processes may affect the overall recovery.

Alcohol exerts a profound impact on neuroinflammation. Although there are some conflicting reports in the literature about the role of alcohol on recovery, the major findings are summarized here. Some animal studies suggest that acute alcohol administration prior to TBI leads to an early reduction in the levels of pro-inflammatory cytokines and chemokines in the injured cortex, hippocampus, and hypothalamus, as well as in the serum shortly after TBI (Goodman et al. 2013; Gottesfeld et al. 2002). Recent studies also have confirmed that acute alcohol intoxication at the time of TBI does not exacerbate the expression of pro-inflammatory cytokines and chemokines at 6 hours post-injury. However, results obtained at a later time point (24 hours) show a sustained mRNA expression of IL-1β, TNF-α, IL-6, and CCL-2 following a lateral fluid percussion injury in rodents that were alcohol-intoxicated at the time of TBI (Teng and Molina 2014). Overall, some preclinical studies suggest that acute alcohol treatment prior to TBI may lead to a suppressed release of pro-inflammatory mediators during the early phase post-injury. Thus, the temporal pattern of neuroinflammatory responses and the impact of alcohol intoxication on neuroinflammatory responses are factors to consider when drawing conclusions on the role of alcohol in modulating the outcome from TBIs.

Because the literature surrounding the relationship between acute alcohol intoxication and response to trauma is conflicting, it is important to consider the pattern of alcohol abuse and the model used in different studies. In general, reports in the literature indicate that chronic alcohol exposure produces immune activation in the brain, inducing an enhanced pro-inflammatory state, as evidenced by the presence of CCL-2 and microglial activation in postmortem brains of human alcoholics (He and Crews 2008). Animal studies show that chronic, intermittent binge alcohol administration to rodents results in increased microglial activation and inflammatory cytokine expression in the cortex and hippocampus (Zhao et al. 2013). In addition, Crews and colleagues (2004) have found that chronic alcohol treatment induces expression of inflammatory cytokines such as TNF-α, which further activates resident glial cells to secrete additional pro-inflammatory cytokines and chemokines, resulting in an increased immune activation in the brain. The overall pro-inflammatory effects of alcohol also have been shown by Guerri and colleagues (2007) who reported alcohol-mediated stimulation of TLR-4 and IL-1 receptor signaling pathways, including extracellular regulated-kinase 1/2 (ERK1/2), stress-activated protein kinase/c-Jun N-terminal kinases (JNK), and p38 mitogen-activated protein kinase (MAPK), as well as the expression of NF-kB, activator protein-1 (AP-1), iNOS, and COX-2 in cultured glial cells (Alfonso-Loeches et al. 2010; Fernandez-Lizarbe et al. 2009). The role of TLR4 has been identified in studies where 5 months of chronic alcohol administration increased glial activation and levels of caspase-3, iNOS, COX-2, and cytokines (IL-1β, TNF-α, and IL-6) in the cerebral cortex of wild-type mice but not in the TLR-4–deficient mice (Alfonso-Loeches et al. 2010). Another mediator of alcohol-mediated neuroinflammation is high-mobility group protein B1 (HMGB1), which has been reported to be increased along with TLR-2, TLR-3, and TLR-4 in postmortem brains of human alcoholics (Alfonso-Loeches et al. 2010). Despite a substantial amount of evidence showing increased neuroinflammatory responses to chronic alcohol exposure, there have not been sufficient preclinical studies performed to determine the combined effect of chronic alcohol consumption and TBI on neuroimmune activation. Because both TBI and alcohol can induce inflammation in the brain, we speculate that the combination of the two events would further accentuate neuroinflammation.

Retrospective studies have revealed that outside of the central nervous system, peripheral organ damage can contribute to the increased mortality rate among TBI patients as a result of cardiovascular, pulmonary, and endocrine dysfunction (Gennarelli et al. 1989; Shavelle et al. 2001). More specifically, TBI patients have an increased incidence of ALI, pulmonary infection, neuroendocrine alterations, and cardiovascular dysfunction during the post-injury period (Vermeij et al. 2013). Although the combined effects of alcohol and TBI and the role of local or systemic immune responses in peripheral organs are understudied, the current knowledge is summarized below (figure 3).

### Pulmonary System

ALI, one of the most common nonneurologic complications following TBI, results from acute pulmonary edema and inflammation and can lead to ARDS (Holland et al. 2003; Johnson and Matthay 2010). ALI is characterized by hypoxemia, loss of lung compliance, and bilateral chest infiltrates (Dushianthan et al. 2011). Development of ALI post-TBI has been associated with increased inpatient mortality following injury and worse long-term neurologic outcome in survivors of TBI (Bratton and Davis 1997; Holland et al. 2003). Post-TBI medical interventions including induced systemic hypertension and mechanical ventilation can result in nonneurogenic ALI (Contant et al. 2001; Lou et al. 2013). Development of neurogenic pulmonary edema (NPE) occurs minutes to hours following TBI and typically resolves within days (Bratton and Davis 1997). The possible underlying factors in NPE are the severity of injury leading to increased intracranial pressure and the subsequent increased circulating catecholamines (Demling and Riessen 1990). TBI also is associated with greater incidence of pulmonary infections than that seen following major surgeries, burn injury, and polytrauma (Dziedzic et al. 2004). Clinical reports indicate that over 40 percent of TBI patients with artificial ventilation develop pneumonia and are four times more likely to die from pneumonia (Harrison-Felix et al. 2006). The increased risk of developing pneumonia post-TBI is potentially attributed in part to a systemic immune response syndrome (SIRS) characterized by increased circulating pro-inflammatory cytokines (TNF-α and IL-6) (Keel and Trentz 2005; Kossmann et al. 1995).

The combined impact of alcohol and TBI on pulmonary infections has been minimally investigated. Although, epidemiological studies have shown that in trauma patients, chronic alcohol abuse can independently increase the risk of ALI and ARDS two- to fourfold (Guidot and Hart 2005). In a prospective study of traumatic injury patients with evidence of acute alcohol intoxication or chronic alcohol abuse, chronic alcohol was associated with increased incidence of pneumonia or respiratory failure as a result of its immunosuppressive effects. However, no significant increase in incidence of pneumonia or respiratory failure and mortality was observed in patients with acute alcohol intoxication with BAC above 100mg/dL (De Guise et al. 2009; Jurkovich et al. 1993). The importance of length and amount of pre-existing alcohol intake and TBI severity may be the key factors in determining a patient’s risk for pneumonia. Taken together, the potential effects of chronic alcohol abuse and TBI could potentiate and further increase immunosuppression or immune dysfunction, thus leading to greater susceptibility for pneumonia, ARDS, and ultimately death.

### Neuroendocrine System

TBI can lead to a variety of neuroendocrine abnormalities, such as gonadotropin deficiency, growth hormone deficiency, corticotrophin deficiency, and vasopressin alterations (Behan and Agha 2007; Powner and Boccalandro 2008). As a result of the mechanical compression to the pituitary gland or disruption of the pituitary stalk, hypopituitarism can occur and corticotrophin insufficiency is commonly observed after TBI (Agha et al. 2004; Cohan et al. 2005). Excessive alcohol use also has been reported to be associated with neuroendocrine dysfunction, notably in the form of altered regulation of hypothalamic–pituitary–adrenal axis (HPA), resulting in a decreased corticotrophin release (Behan and Agha 2007; Helms et al. 2014). Therefore, it is possible that the combination of alcohol and TBI-induced HPA dysfunction can lead to a dampened cortisol release, which may have an impact on the immune system. Interestingly, a hyperadrenergic state marked by elevated levels of catecholamines can occur after TBI, and alcohol intoxication at the time of TBI has been shown to blunt the sympatho-adrenal activation in a dose-dependent manner (Woolf et al. 1990). Vasopressin has been suggested to play a role in blood brain barrier disruption, edema formation, and the production of pro-inflammatory mediators after TBI (Szmydynger-Chodobska et al. 2010). Vasopressin abnormalities leading to diabetes insipidus or the syndrome of inappropriate anti-diuretic hormone (SIADH) frequently are observed after TBI (Behan and Agha 2007), and acute alcohol intoxication is known to alter AVP release (Taivainen et al. 1995). Whether alcohol intoxication at the time of TBI or during the recovery period from TBI further dysregulates these neuroendocrine mechanisms remains to be examined.

### Cardiovascular System

Cardiovascular complications including slow heart rate (i.e., bradycardia), hypotension, electrocardiographic changes, arrhythmias, and increased circulating cardiac enzymes have been reported following TBI (Bourdages et al. 2010; Wittebole et al. 2005). Chronic alcohol abuse alone can lead to alcoholic cardiomyopathy and potentially heart failure (Skotzko et al. 2009), and the underlying etiology has been reviewed (Lang et al. 2005). Several studies by Zink and colleagues (1998*a*,*b*, 2006) focused on the combined effects of acute alcohol intoxication on hemorrhagic shock and TBI in swine, showing decreased survival time, lowered MABP, and reduced cerebral perfusion pressure, which may worsen secondary brain injury. These studies did not investigate alterations in immune function or expression and levels of immune modulators or their actions on cardiovascular function. Overall, the post-TBI cardiovascular complications, including vascular function, have been understudied in both clinical and experimental models of TBI. More specifically, the combined impact of alcohol, TBI, and immune alterations on cardiovascular dysfunction and disease progression has not been examined. A possible prediction is that chronic alcohol-induced immunosuppression would worsen post-TBI cardiovascular complications; and in chronic alcoholics, dilated cardiomyopathy may compound TBI-related cardiovascular complications increasing morbidity and mortality.

## Summary

The deleterious effects of alcohol on the immune system in three traumatic injuries are discussed in this review and are summarized in figures 1, 2, and 3. It is evident that, independently, acute or chronic alcohol consumption and traumatic injury negatively modulate the immune system, and the end result is an uncontrolled release of inflammatory mediators. The most important message of this review is the accumulation of evidence that alcohol combined with traumatic injury can significantly affect morbidity and mortality through disruption in host immune responses. Following burn injury, for instance, the risk for infection is greatly increased because of increased gut permeability and increased pro-inflammatory cytokine expression in the lungs (figure 1). Alcohol use following hemorrhage can increase inflammation and oxidative stress in the gut while decreasing lung barrier function and subsequently increasing susceptibility to infection (figure 2). In the central nervous system, alcohol use following TBI can increase neuroinflammation and prolong the recovery period (figure 3). Overall this information is important, because it provides a wealth of evidence that alcohol combined with trauma is a dramatic and preventable cause of increased morbidity and mortality following injury. Mechanistically, two common pro-inflammatory cytokines that are consistently upregulated in all burn injury, hemorrhagic shock, and TBI are TNF-α and IL-6. A fuller understanding of their temporal pattern of expression and downstream effects requires further investigation. Although the studies described in this review have generated important information on the impact of alcohol combined with different types of traumatic injury, and the resultant adverse effects on the immune system, further preclinical and clinical studies to dissect the complex cascade of immunomodulation following injury are necessary. Specifically, further investigation is warranted to determine the underlying mechanisms involved in immune modulation by acute or chronic alcohol intake and the effects on (1) metabolism and the cardiovascular system following burn, (2) the neuroendocrine system following hemorrhagic shock, and (3) neuroinflammation and the neuroendocrine system following traumatic injury. The responses of the immune system to these inflammatory stimuli are variable and appear to be dependent on the severity of the injury, comorbidities, and the level of alcohol intoxication. Thus, it is necessary to systemically address these variables for translational research to identify potential therapeutic strategies. Furthermore, therapeutic targets for immunomodulation and attenuation of tissue injury in intoxicated and injured patients are likely to reduce morbidity and mortality and improve post-injury quality of life among these patients.
